# The neutrophil to lymphocyte ratio (NLR) and the presence of large nodal mass are independent predictors of early response: A subanalysis of the prospective phase II PET‐2‐adapted HD0607 trial

**DOI:** 10.1002/cam4.3396

**Published:** 2020-11-06

**Authors:** Alessandra Romano, Chiara Pavoni, Francesco Di Raimondo, Corrado Tarella, Simonetta Viviani, Andrea Rossi, Caterina Patti, Marco Picardi, Maria Cantonetti, Giorgio La Nasa, Livio Trentin, Silvia Bolis, Valerio Zoli, Paolo Gavarotti, Paolo Corradini, Michele Cimminiello, Corrado Schiavotto, Guido Parvis, Roberta Zanotti, Guido Gini, Andrés J. M. Ferreri, Piera Viero, Stephane Chauvie, Alberto Biggi, Alessandro Massimo Gianni, Andrea Gallamini, Alessandro Rambaldi

**Affiliations:** ^1^ Dipartimento di Specialità medico‐Chirurgiche, CHIRMED Sezione di Ematologia Università degli studi di Catania Catania Italy; ^2^ Ematologia Azienda Socio Sanitaria Territoriale Papa Giovanni XXIII Bergamo Italy; ^3^ Onco‐Hematology Unit Istituto Europeo di Oncologia Milan Italy; ^4^ Università degli Studi di Milano Milano Italy; ^5^ Ematologia e onco‐ematologia pediatrica Fondazione IRCSS Istituto Nazionale dei Tumori Milan Italy; ^6^ Ematologia Azienda Villa Sofia‐Cervello Palermo Italy; ^7^ Ematologia Università Federico II Napoli Italy; ^8^ Oncoematologia Policlinico Tor Vergata Roma Italy; ^9^ Ematologia Ospedale R.Binaghi Cagliari Italy; ^10^ Ematologia Dipartimento di Medicina Università di Padova Padova Italy; ^11^ Ematologia Ospedale San Gerardo Monza Italy; ^12^ Ematologia Ospedale S. Camillo Roma Italy; ^13^ Ematologia Universitaria Azienda Ospedaliera Universitaria Città della Salute e della Scienza di Torino Italy; ^14^ Dipartimento di oncologia‐ematologia Università degli Studi di Milano Milano Italy; ^15^ Divisione Universitaria di Ematologia Ospedale San Carlo Potenza Italy; ^16^ Ematologia Presidio Ospedaliero S. Bortolo Vicenza Italy; ^17^ Divisione Universitaria Medicina Interna AO San Luigi Orbassano Italy; ^18^ Divisione di Medicina Unità di Ematologia Azienda Ospedaliera Universitaria Integrata Verona Italy; ^19^ Divisione Universitaria di Ematologia Nuovo Ospedale Torrette Ancona Italy; ^20^ Unità di Ricerca Clinica Linfomi IRCSS Ospedale San Raffaele Milano Italy; ^21^ Ematologia Ospedale dell'Angelo Mestre Italy; ^22^ Medicina Nucleare Azienda Ospedaliera Santa Croce e Carle Cuneo Italy; ^23^ Department recherch e innovation et statistique Centre A. Lacassagne Nice France

**Keywords:** biomarkers, hodgkin lymphoma, neutrophil to lymphocyte ratio, PET‐2

## Abstract

**Background:**

The neutrophil to lymphocyte ratio (NLR) and the lymphocyte to monocyte ratio (LMR) can reflect both the myeloid dysfunction and T‐cell immune suppression and have prognostic significance.

**Methods:**

In 771 newly diagnosed advanced‐stage Hodgkin Lymphoma (HL) patients we evaluated the baseline values of NLR and LMR as predictors of clinical outcome. According to the multicenter prospective phase II GITIL‐HD0607 trial, all patients received two ABVD courses and if PET‐2 negative received four additional ABVD cycles while if PET‐2‐positive patients were randomized to either BEACOPP escalated (Be) plus BEACOPP baseline (Bb) (4 + 4 courses) or Be + Bb (4 + 4) and Rituximab. PET scans were centrally reviewed by an expert panel by Blinded Independent Central Review.

**Results:**

Higher NLR and lower LMR were associated with a PET‐2 positivity and failure to achieve long‐term disease control, respectively. By univariate and multivariate analysis, large nodal mass (>7 cm), IPS ≥ 3, NLR > 6 were strong independent predictors of early PET‐2 response after ABVD. Only NLR > 6 and IPS ≥ 3 were strong independent predictors of outcome at diagnosis; however, when PET‐2 status was added, only PET‐2‐positive status and IPS ≥ 3 were independent predictors of PFS. Focusing on PET‐2‐negative patients, those with NLR > 6 had an inferior 3‐year PFS compared to patients with NLR ≤ 6 (84% vs 89% months, *P* = .03).

**Conclusion:**

In advanced‐stage HL patients treated with a PET‐2‐driven strategy, IPS ≥ 3 and NLR > 6 are independent predictors of outcome at diagnosis while the presence of large nodal mass, IPS ≥ 3, and NLR > 6 at diagnosis are independent predictors of early ABVD response.

## Graphical abstract

In the setting of newly diagnosed advanced‐stage HL patients, the presence of large nodal mass, IPS ≥ 3, and NLR > 6 at diagnosis are independent predictors of early ABVD response. IPS ≥ 3 and NLR > 6 are also useful predictors of outcome in PET‐2‐negative patients.

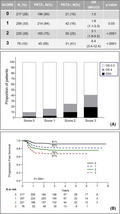

## 1 INTRODUCTION

Immune accessory cells of the microenvironment play a major part in the development and progression of Hodgkin Lymphoma (HL).^1^ The activity of microenvironment is captured by ^18^F‐fuoro‐2‐deoxy‐glucose positron emission tomography (FDG‐PET) that, in fact, is a surrogate test of tumor chemosensitivity, and if positive can indicate the persistence of high glycolytic activity in the microenvironment.^2^, ^3^, ^4^

A positive uptake in FDG‐PET, performed early after the first two cycles of chemotherapy (PET‐2),^5^, ^6^, ^7^, ^8^ is to date the major predictor factor in HL^9^, ^10^ and it has been exploited to base a risk‐adapted strategy.^11^, ^12^, ^13^, ^14^ Our group recently described the long‐term results of the GITIL/FIL HD0607 trial, showing that the PET‐2‐driven switch from ABVD (doxorubicin, bleomycin, vinblastine, dacarbazine) to escalated BEACOPP (bleomycin, etoposide, doxorubicin, cyclophosphamide, vincristine, procarbazine, and prednisone) is feasible and effective in high‐risk patients with advanced‐stage HL.^15^

PET‐2 is more sensitive than the current prognostic model International Prognostic Score (IPS) ^16^ in predicting poor outcome.^5^ IPS, developed on the basis of retrospective international series of HL patients treated before 1992, has limited clinical utility because only 19% of patients with scores 4 and 5 had a probability of 7‐year progression‐free survival (PFS)< 50%.^16^, ^17^

However, PET‐2 information, as other therapy‐restricted predictive factors, is available only during treatment and it is likely that biological events responsible of chemoresistance may be activated as early as after two cycles of treatment in nonresponder patients. Thus, the availability of a biomarker at diagnosis able to address high‐risk patients to a more aggressive risk‐adapted strategy is an unmet clinical need.

In the attempt to identify treatment independent prognostic factor at diagnosis, and not during treatment, novel insights in HL biology have translated in emerging prognostic factors,^18^, ^19^, ^20^, ^21^, ^22^ not always validated in prospective clinical trials.^7^

We and others have shown that both neutrophil‐like and monocyte‐like myeloid‐derived suppressor cells (MDSCs) have an important prognostic role because they exert a strong immunosuppressive effect on the T‐cell function, reducing their ability of immune surveillance and therefore favoring neoplastic progression. Both neutrophil‐like MDSC and neutrophils are elevated in the peripheral blood of HL patients, secrete arginase, which confers immunosuppressive properties, and are positively related to tumor‐associated macrophages (TAM).^19^, ^20^

Since the amount of TAM in the diagnostic biopsy^23^ is predictive of outcome, several groups investigated the prognostic role of neutrophil to lymphocyte ratio (NLR) and the lymphocyte to monocyte ratio (LMR) as surrogate markers of the complex network of myeloid cells and cytokines in HL microenvironment.^20^, ^24^ Indeed, an increase in neutrophils is common in HL and is associated with a negative prognosis^20^, ^25^ even if not included in the IPS.^16^ HL neutrophils are dysfunctional and reflect the amount of both granulocytic and monocytic myeloid‐derived suppressor cells in peripheral blood and tumor‐associated macrophages in the lymph nodes.^20^ Lymphopenia, defined as < 600 cells/μL or < 8% of the WBC, is recognized by the IPS as an adverse prognostic factor.^16^

High NLR and low LMR have been reported in several retrospective series (Table S1) as negative prognostic factors for both PFS and OS in HL,^26^, ^27^, ^28^, ^29^, ^30^, ^31^, ^32^, ^33^, ^34^ but limited data are available in PET‐2 era.^6^, ^35^, ^36^ Thus, we took advantage of the prospective multicenter phase 2 HD0607 trial to test the clinical meaning of NLR and LMR in patients treated upfront with a risk‐adapted strategy, based on a blinded independent central review of PET‐2.

## 2 METHODS

### 2.1 Study design

We reviewed the clinical research forms (CRF) of 780 patients with newly diagnosed, advanced‐stage HL enrolled in the prospective, open‐label randomized phase II trial HD0607 which aimed to improve the 3‐year PFS of advanced‐stage HL patients switching from ABVD to escalated BEACOPP when an early interim PET proved positive.^15^ White blood cells differential counts missed in nine cases, thus 771 subjects were eligible for the study.

The HD0607 trial (NCT00795613, Eudract code 2007‐007168‐94 at ClinicalTRials.gov) was conducted in accordance with the International Conference on Harmonization for Good Clinical Practice guidelines and the Declaration of Helsinki. Before enrollment, all patients gave their written informed consent, as per the Italian Pharmacology Agency (AIFA) and the ethics committees of all the participating centers approvals.

All patients received two cycles of ABVD (administered at standard doses on days 1 and 15), followed by an early interim PET (PET‐2) re‐evaluation. Based on a blinded independent central review, PET‐2 was reported as negative in presence of a Deauville score of 1‐3, while PET scans with a score 4‐5 were reported as positive.

PET‐2‐positive patients were treated with BEACOPP, with or without the addition of Rituximab, 375 mg/m^2^ iv, given on the day 1 of each BEACOPP course. Patients with a negative PET‐2 continued their standard ABVD treatment for a total of six courses. Among them, those with a large nodal mass at baseline and a negative interim and final PET at restaging were randomized to receive consolidation radiotherapy (RT, 30 Gy) on the site where a large nodal mass was detected at diagnosis or no further treatment.

Per protocol procedures, complete blood count and routine biochemical examinations were taken before start treatment. White blood cell differential counts were determined by electrical impedance method in automatic blood counter devices in each center.

**FIGURE 1:**
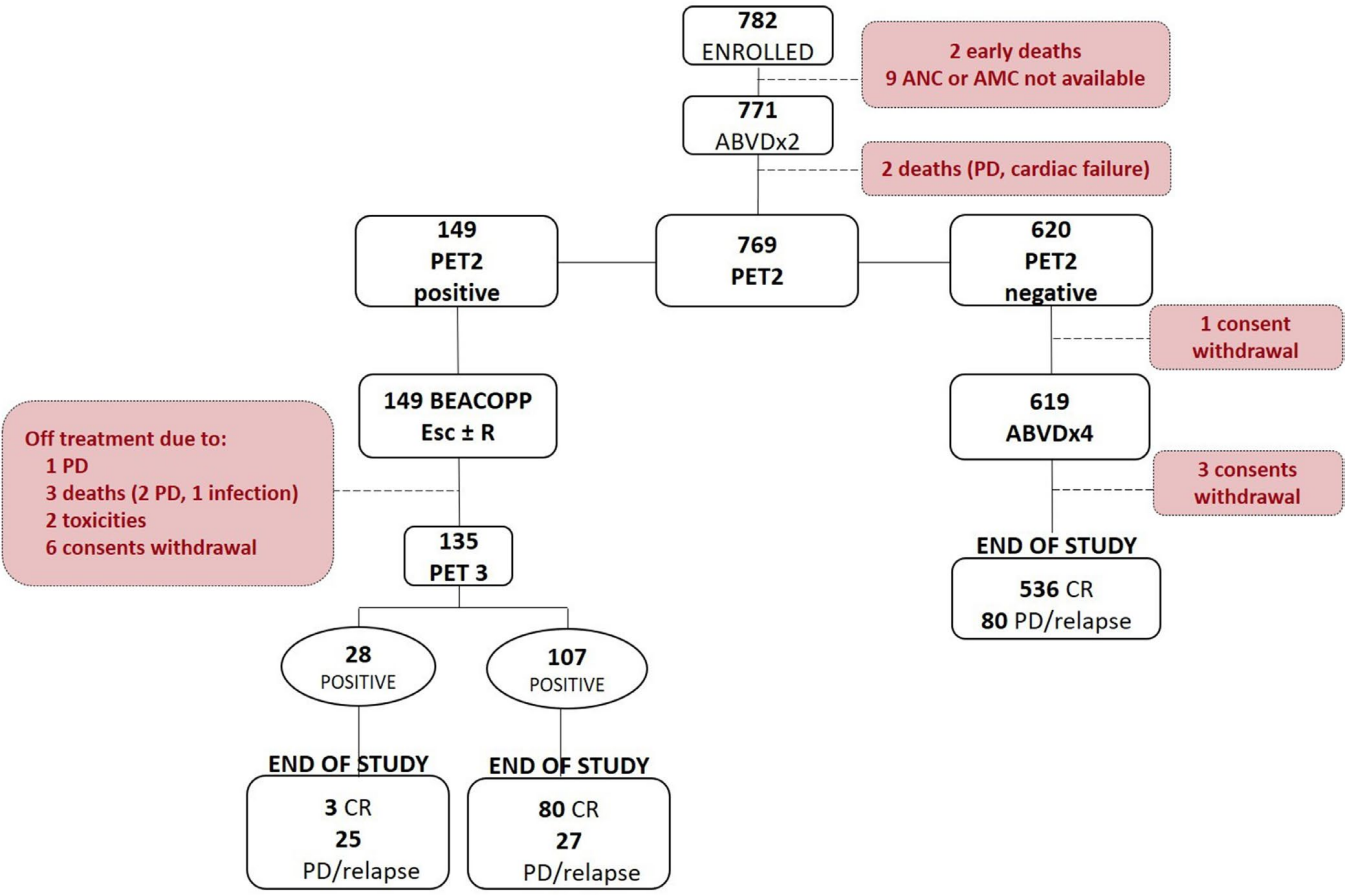
Allocation of patients evaluated in the study

The entire workflow of registered patients across the study protocol, the interim PET, NLR, and LMR results and treatment outcomes are shown in Figure 1, while patients’ characteristics at baseline and clinical outcome according to NLR and LMR are summarized in Table 1.

**TABLE 1:** Characteristics at baseline and clinical outcome of 771 advanced‐stage HL patients enrolled in the HD0607 trial, according to NLR and LMR at diagnosis

	All patients, N = 771	NLR ≤ 6, N = 418	NLR > 6, N = 353	*P*‐ value	LMR ≤ 2, N = 399	LMR > 2, N = 372	*P*‐ value
Age (y)							
Median (range)	31 (14‐60)	31 (14‐60)	30 (16‐60)	.0674	31 (16‐60)	31 (14‐60)	.6502
<45, n(%)	637 (82.6)	332 (79.4)	305 (86.4)	*.0109*	334 (83.7)	303 (81.5)	.4084
≥45, n(%)	134 (17.4)	86 (20.6)	48 (13.6)		65 (16.3)	69 (18.5)	
Sex, n(%)							
Female	394 (51.1)	197 (47.1)	197 (55.8)	*.0163*	185 (46.4)	209 (56.2)	*.0064*
Male	377 (48.9)	221 (52.9)	156 (44.2)		214 (53.6)	163 (43.8)	
Ann Arbor Stage, n(%)							
IIB	275 (35.7)	146 (34.9)	129 (36.5)	.3639	135 (33.8)	140 (37.6)	.0637
III	249 (32.3)	144 (34.4)	105 (29.7)		121 (30.3)	128 (34.4)	
IV	247 (32)	128 (30.6)	119 (33.7)		143 (35.8)	104 (28)	
Histology							
Nodular sclerosis	624 (81)	318 (76.3)	306 (86.7)	*.0002*	326 (81.9)	298 (80.1)	.5238
Other	146 (19)	99 (23.7)	47 (13.3)		72 (18.1)	74 (19.9)	
B Symptoms, n(%)							
No	147 (19.1)	102 (24.4)	45 (12.7)	*<.0001*	56 (14)	91 (24.5)	*.0002*
Yes	624 (80.9)	316 (75.6)	308 (87.3)		343 (86)	281 (75.5)	
ALC, median (range)	1.4 (0.2‐15.6)	1.7 (0.4‐4.6)	1.2 (0.2‐15.6)	*<.0001*	1.1 (0.2‐11.3)	1.8 (0.2‐15.6)	*<.0001*
ANC, median (range)	8.1 (0.3‐117.9)	6.3 (0.3‐18.2)	10.7 (2.3‐117.9)	*<.0001*	8.5 (1.3‐72.4)	7.2 (0.3‐117.9)	*<.0001*
AMC, median (range)	0.7 (0‐5.6)	0.7 (0.1‐1.9)	0.8 (0‐5.6)	*.0005*	0.8 (0.2‐5.6)	0.6 (0‐4.3)	*<.0001*
NLR, median (range)	5.7 (0.3‐85.6)	4 (0.3‐6)	8.8 (6‐85.6)	*<.0001*	7.6 (1.5‐85.6)	4.2 (0.3‐27)	*<.0001*
LMR, median (range)	2 (0.2‐22)	2.5 (0.8‐16.5)	1.5 (0.2‐22)	*<0.0001*	1.5 (0.2‐2)	2.8 (2‐22)	*<.0001*
Large nodal mass, n(%)							
≤7 cm	461 (59.8)	294 (70.3)	167 (47.3)	*<.0001*	210 (52.6)	251 (67.5)	*<.0001*
>7 cm	310 (40.2)	124 (29.7)	186 (52.7)		189 (47.4)	121 (32.5)	
Bone marrow biopsy, n(%)							
Negative	705 (94.4)	375 (92.4)	330 (96.8)	0.0092	368 (95.3)	337 (93.4)	.2392
Positive	42 (5.6)	31 (7.6)	11 (3.2)		18 (4.7)	24 (6.6)	
IPS score, n(%)							
<3	507 (65.7)	311 (74.4)	196 (55.5)	*<.0001*	222 (55.6)	285 (76.6)	*<.0001*
≥3	264 (31.4)	107 (25.6)	157 (44.5)		177 (44.4)	87 (23.4)	
PET‐2 Deauville Score, n (%)							
0‐3	620 (80.6)	356 (85.4)	264 (75)	*.0008*	310 (77.9)	310 (83.6)	.1296
4	100 (13)	44 (10.6)	56 (15.9)		58 (14.6)	42 (11.3)	
5	49 (6.4)	17 (4.1)	32 (9.1)		30 (7.5)	19 (5.1)	
Response to treatment							
CR	665 (88.7)	375 (91.9)	290 (84.8)	*.0022*	334 (86.1)	331 (91.4)	*.0208*
Progressive disease	85 (11.3)	33 (8.1)	52 (15.2)		54 (13.9)	31 (8.6)	

Note

Differences in categorical parameters were evaluated using chi‐squared of Fisher's exact test, as appropriate. Differences in continuous parameters were evaluated using Mann‐Whitney U test. Significant values are in italic.

Abbreviations: ALC, absolute lymphocyte count/mmc; AMC, absolute monocyte count/mmc; ANC, absolute neutrophil count/mmc; IPS, International Prognostic Score; LMR, lymphocyte to monocyte ratio; NLR neutrophil to lymphocyte ratio.

### 2.2 Statistical analysis

Descriptive data are presented through median with range for continuous variables and frequency with percentage for categorical variables. Pairwise comparisons were performed using the Mann‐Whitney test for continuous variables and the Pearson's chi‐squared test or the Fisher's exact test for categorical variables. Receiver operating characteristic (ROC) analysis was performed to assess the utility of NLR and LMR to predict treatment failure (progression, relapse, or death) at diagnosis and to confirm the best thresholds identified by previous retrospective series in the field (2).

Survival outcomes were estimated using the Kaplan‐Meier method and the log‐rank test was applied to test differences between groups. PFS was measured from the date of registration to the date of first appearance of disease progression, relapse, or death for any cause or, whichever came first, to the date of the last follow‐up visit; positive status of PET‐2 was not considered as an event. Overall survival (OS) was measured from the date of registration to the date of death for any cause or to the date of the last follow‐up visit. Predictive factors of PET‐2‐positive status were assessed with logistic regression, while Cox proportional hazard models were performed to assess factors that were predictive of PFS and OS. Proportional hazard assumption was verified for all estimated models. All reported p‐values were two sided and the conventional 5% significance level was fixed. Statistical analysis was performed using SAS (version 9.4) and R (version 3.5.0).

## 3 RESULTS

### 3.1 NLR and LMR values at diagnosis

In the HD0607 trial, 782 newly diagnosed advanced‐stage HL patients have been enrolled^15^; NLR and LMR were available for 771 patients (Figure 1). The median age was 31 years (range 14‐60), and half patients were females (Table 1). The medians of absolute neutrophil (ANC), lymphocyte (ALC), and monocyte (AMC) counts were 8.1 (range 0.3‐117.9) × 10^3^/μL, 1.4 (range 0.2‐15.6) × 10^3^/μL, and 0.7 (range 0‐5.6) × 10^3^/μL, respectively. Thus, median NLR and LMR at diagnosis were 5.7 (range 3.8‐8.3) and 2.0 (range 1.4‐2.8), respectively, both higher than in healthy subjects as previously reported.^35^

NLR was increased and LMR was reduced in patients with B symptoms (*P* < .0001), large nodal mass > 7 cm (*P* < .0001), and IPS score higher than 3 (*P* < .0001) (Table S2). Females younger than 45 years had higher NLR than males (*P* = .0022) while LMR was lower in males (*P* = .014, Table S2). There were no significant differences of NLR and LMR based on Ann Arbor stage.

**FIGURE 2:**
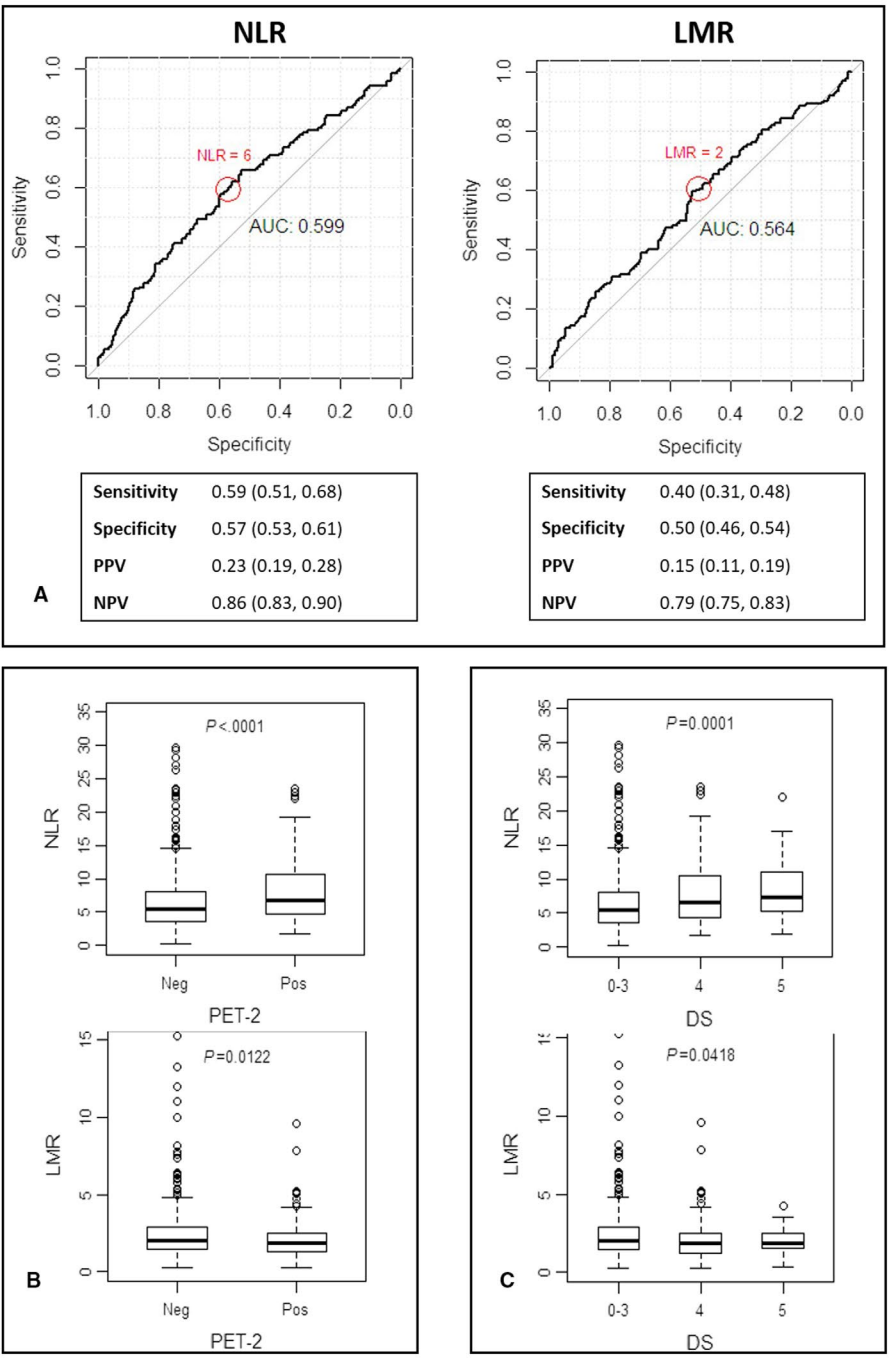
NLR and LMR are associated with clinical variables at diagnosis and outcome in 771 advanced‐stage HL patients enrolled in the HD0607 trial. A, ROC analysis to evaluate specificity and sensitivity of NLR and LMR. B, Pretreatment median NLR and LMR were increased in patients with positive PET‐2 (A). C. Baseline NLR and LMR and PET‐2‐positive status according to the Deauville score (DS) 5 carrying new lesion or DS 4 (residual activity)

ROC analysis identified the more accurate threshold value to predict treatment outcome (Figure 2A), in terms of 3‐year PFS for NLR and LMR in 6 and 2, respectively (Figure 2). The AUC for NLR was 0.6 (95% CI = 0.54‐0.65); using NLR = 6 as cutoff, treatment failure was identified with sensitivity of 59% (95% CI = 51%‐68%) and specificity of 57% (95% CI = 53%‐61%), with high‐negative predictive value of 86% (95% CI = 83%‐90%), but with a low‐positive predictive value 23% (95% CI = 19%‐28%).

Similarly, the AUC for LMR was 0.56 (95% CI = 0.51‐0.62); LMR < 2 could identify treatment failure with sensitivity of 40% (95% CI = 31%‐48%) and specificity of 50% (95% CI = 46%‐54%), with high‐negative predictive value equal to 79% (95% CI = 75%‐83%).

### 3.2 NLR and LMR can predict clinical outcome

Two patients died during the first two courses of ABVD for disease progression in one case and cardiac failure in the other. According to protocol procedure, 769 patients underwent interim PET‐2 scanning: 149 (19.4%) patients had a positive PET‐2 (100 with score 4 and 49 with score 5) and 620 (80.6%) patients had negative PET‐2. PET‐2‐positive patients were allocated to the escalated BEACOPP program. As per medical decision, one patient was not randomized and received escalated BEACOPP. During the first four escalated BEACOPP, six patients withdrew their consent and underwent alternative treatment, three patients died due to disease progression (n = 2) and infection (n = 1), one patient progressed while two patients stopped treatment due to toxicity. A third PET evaluation was performed in 135 patients and a disease progression was registered in 27 of 107 PET‐3 negative as compared to 25 of 28 PET‐3 positive. In the cohort of PET‐2‐negative patients, 619 continued with four additional ABVD and 536 (86%) achieved a durable CR; 80 patients (13%) had a treatment failure and four patients withdrew consent (Figure 1).

**FIGURE 3:**
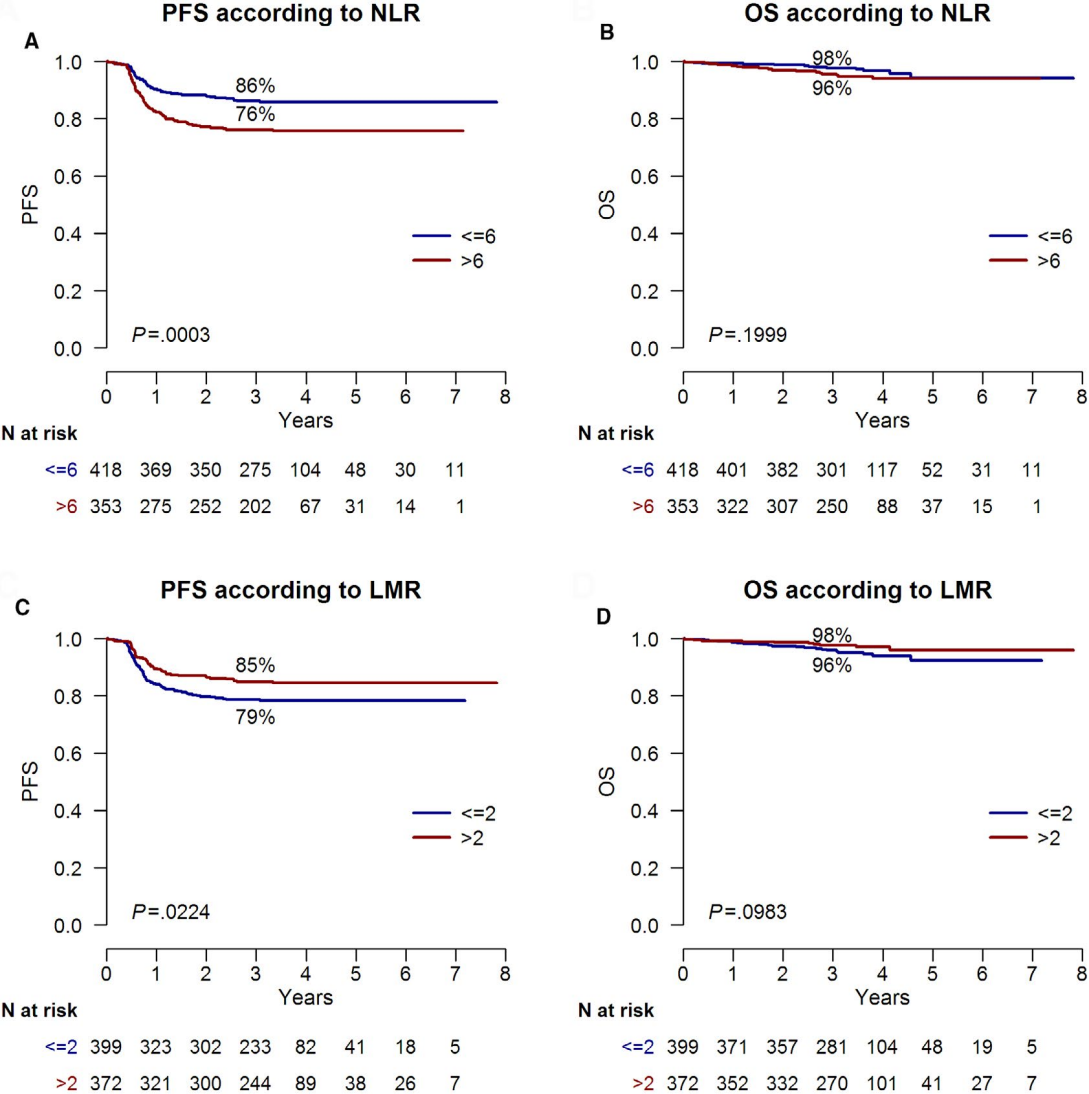
Progression‐free survival and overall survival in newly diagnosed HL patients according to pretreatment NLR (A and B) and LMR (C and D). Progression‐free survival (A) and overall survival (B) in 771 newly diagnosed HL patients based on NLR > 6. Progression‐free survival (C) and overall survival (D) in 771 newly diagnosed HL patients based on LMR < 2

In an intention‐to‐treat analysis, after a median follow‐up of 3.6 years, the 3‐year PFS and OS for all 771 patients were 82% and 97%, respectively. Patients carrying on NLR > 6 or LMR < 2 at baseline had inferior 3‐year PFS (76% (95%CI, 72%‐81%) vs 86% (95%CI, 83%‐90%), *P* = .0003, Figure 3A; 79% (95%CI, 75%‐83%) vs 85% (95%CI,81%‐89%), *P* = .02, Figure 3C, respectively). We evaluated NLR and LMR as predictors of outcome at baseline or in association to PET‐2 status (Table 2, Table S3). Predictors at diagnosis of treatment failure were NLR (*P* = .0003), LMR (*P* = .02), and IPS (*P* < .0001) in univariate analysis, but only NLR > 6 (*P* = .03) and IPS ≥ 3 (*P* = .0001) were independent factors in multivariable analysis (Table 2). However, when we added PET‐2 status among clinical parameters predictors of outcome, only PET‐2 status (*P* < .0001) and IPS ≥ 3 (*P* = .0001) were independent factors in multivariate analysis (Table 2). Neither NLR or LMR at baseline were predictors of OS (Figure 3B‐D).

**TABLE 2:** Multivariable analysis of progression‐free survival in advanced‐stage HL patients enrolled in the HD0607 trial, according to clinical predictors, including PET‐2 status

	Clinical variables available at baseline				All clinical variables (including PET‐2 status)			
Predictive factors	Multivariable without LMR		Multivariable without NLR		Multivariable without LMR		Multivariable without NLR	
	HR (95% CI)	*P*‐value	HR (95% CI)	*P*‐value	HR (95% CI)	*P*‐value	HR (95% CI)	*P*‐value
NLR								
≤6	1				1			
>6	1.48 (1.04‐2.12)	*.03*			1.37 (0.96‐1.97)	.0849		
LMR								
≤2			1				1	
>2			0.87 (0.61‐1.24)	.44			0.89 (0.62‐1.28)	.5296
PET‐2								
Negative					1		1	
Positive					3.37 (2.37‐4.8)	*<.0001*	3.45 (2.43‐4.9)	*<.0001*
B Symptoms								
Absent	1		1		1		1	
Present	1.09 (0.68‐1.75)	.73	1.14 (0.71‐1.82)	.60	1.19 (0.74‐1.91)	.4830	1.22 (0.76‐1.96)	.4112
Large nodal mass								
≤7 cm	1		1		1		1	
>7 cm	1.24 (0.88‐1.75)	.22	1.33 (0.95‐1.87)	.11	1.06 (0.75‐1.51)	.7382	1.12 (0.79‐1.59)	.5133
IPS (N)								
<3	1		1		1		1	
≥3	2.30 (1.63‐3.25)	*<.0001*	2.39 (1.69‐3.38)	*<.0001*	1.98 (1.4‐2.81)	*.0001*	2.03 (1.43‐2.89)	*.0001*

Note

Significant *P* values are in italic.

Abbreviations: 95% CI, 95% confidence interval; HR, Hazard ratio; IPS, International Prognostic Score; LMR, lymphocyte to monocyte ratio; NLR, neutrophil to lymphocyte ratio.

In the subset of 624 patients carrying on nodular sclerosis histotype, NLR > 6 (*P* = .01), IPS >= 3 (*P* = .0002) were independent predictors of PFS at baseline, and early during treatment, remaining independent from PET‐2 positivity, as shown in detail in Table 3.

**TABLE 3:** Multivariable analysis of progression‐free survival in 624 advanced‐stage HL patients, nodular sclerosis histotype, enrolled in the HD0607 trial, according to clinical predictors, including PET‐2 status

Predictive factors	Clinical variables available at baseline				All clinical variables (including PET‐2 status)			
	Multivariable without LMR		Multivariable without NLR		Multivariable without LMR		Multivariable without NLR	
	HR (95% CI)	*P*‐value	HR (95% CI)	*P*‐value	HR (95% CI)	*P*‐value	HR (95% CI)	*P*‐value
NLR								
≤6	1				1			
>6	1.74 (1.13‐2.66)	*.01*			1.63 (1.05‐2.51)	*.03*		
LMR								
≤2			1				1	
>2			0.88 (0.58‐1.32)	.53			0.88 (0.58‐1.33)	.54
PET‐2								
Negative					1		1	
Positive					3.2 (2.12‐4.83)	*<.0001*	3.34 (2.21‐5.05)	*<.0001*
B Symptoms								
Absent	1		1		1		1	
Present	0.97 (0.57‐1.66)	.93	1.05 (0.62‐1.78)	.86	1.08 (0.64‐1.85)	.77	1.15 (0.67‐1.95)	.61
Large nodal mass								
≤7 cm	1		1		1		1	
>7 cm	1.06 (0.71‐1.58)	.79	1.19 (0.8‐1.76)	.38	0.88 (0.59‐1.33)	.55	0.97 (0.65‐1.45)	.90
IPS (N)								
<3	1		1		1		1	
≥3	2.12 (1.43‐3.16)	*.0002*	2.29 (1.54‐3.41)	*<.0001*	1.81 (1.21‐2.7)	*.004*	1.9 (1.26‐2.85)	*.002*

Note

Significant *P* values are in italic.

Abbreviations: 95% CI, 95% confidence interval; HR, Hazard ratio; IPS, International Prognostic Score; LMR, lymphocyte to monocyte ratio; NLR, neutrophil to lymphocyte ratio.

### 3.3 NLR and LMR at diagnosis can predict interim PET‐2 status

Median NLR at diagnosis was higher in PET‐2‐positive than PET‐2‐negative patients (6.8, IQR 4.7‐10.7 vs 5.5, IQR 3.6‐8.0, *P* < .0001), and, among PET‐2‐positive patients, NLR was higher in case of Deauville Score (DS) 5 than 4 (7.3, IQR 5.2‐11.0 vs 6.5, IQR 4.3‐10.5, *P* < .0001), as shown in Table S2 and Figure 2B.

Median LMR at diagnosis was lower in PET‐2‐positive than PET‐2‐negative patients (1.8, IQR 1.3‐2.5 vs 2.0, IQR 1.5‐2.9, *P* = .01), without any difference between Deauville Score (DS) 5 or 4 (Table S2 , Figure 2C).

Predictors at baseline of PET‐2‐positive status were NLR (*P* = .0003), LMR (*P* = .047), large nodal mass (*P* < .0001), and IPS (*P* < .0001) in univariate analysis, but only NLR > 6 (*P* = .042), large nodal mass (*P* = .0001), and IPS ≥ 3 (*P* = .0001) were independent factors in multivariable analysis (Table S4).

**FIGURE 4:**
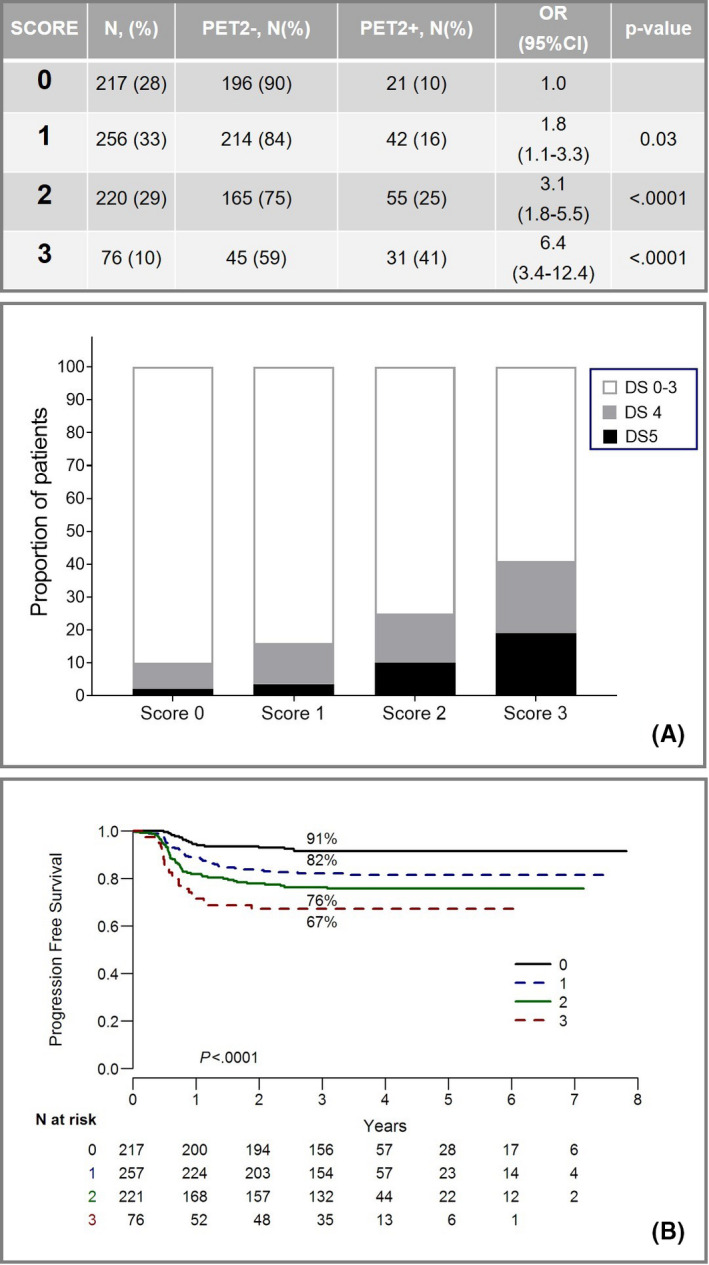
Outcome prediction based on the combination of IPS, NLR, and presence of large nodal mass at diagnosis

In the attempt to predict the overall clinical outcome at diagnosis, we combined the independent predictors of PET‐2‐positive status in a score, given 1 point each to IPS ≥ 3, large nodal mass > 7 cm, and NLR > 6. In all, 28% (N = 217) of the patients were classified in the very‐low risk group (score 0), 33% (N = 256) standard risk (score 1), 29% (N = 220) as high‐risk (score 2), and 10% (N = 76) very‐high risk (score 3). Score 0 was enriched of PET‐2‐negative cases, while score 3 was enriched of PET‐2‐positive cases, with a progressive enrichment of cases with Deauville score 5 (Figure 4A). The 3‐year PFS estimates were 91%, 82% 76%, and 67% for very‐low, standard‐risk, high‐risk, and very‐high risk groups, respectively (Figure 4B).

### 3.4 NLR and LMR can predict clinical outcome in PET‐2‐negative HL patients

For PET‐2‐positive and PET‐2‐negative patients, the 3‐year PFS was 60% and 87% and the 3‐year OS was 89% and 99%, respectively.

We therefore analyzed NLR > 6 and LMR < 2 to predict clinical outcome in PET‐2‐negative patients. Patients carrying NLR > 6 had an inferior 3‐year PFS compared to patients with NLR ≤ 6 (84% vs 89% months, *P* = .03), while there was no statistical difference based on LMR status. As shown in Table S5, predictors at baseline of 3‐year PFS in 628 PET‐2‐negative patients were high NLR (*P* = .043) and high‐score IPS (*P* = .002), but only IPS ≥ 3 maintained its prognostic significance in multivariable analysis (*P* = .0007). When we considered the contribution of each IPS variable, we found that male sex, WBC ≥ 15 000/mm^3^, lymphocyte < 600/mm^3^ or < 8% of WBC, and large nodal mass > 7 cm retained their prognostic meaning in predicting PET‐2 positivity in both univariate and multivariable analysis (Table S5).

## 4 DISCUSSION

In this study, we show that NLR > 6 and, with a minor role, LMR < 2, calculated at diagnosis, have a negative prognostic meaning in advanced HL treated with a PET‐2‐dependent approach. These parameters are associated with negative features of disease such as B symptoms, large nodal mass, and IPS score ≥ 3. They also can predict a positive PET‐2 and a worst clinical outcome at least in term of PFS. In addition, we observed that the negative prognostic significance of NLR > 6 could apply also in patients who achieve a PET‐2‐negative status, so far considered at good prognosis.

In our series, NLR > 6 was also an independent predictor of PFS in HL nodular sclerosis, confirming what previously seen in a retrospective cohort in the pre‐PET era^26^ and supporting the preclinical evidence that histological subtype can be associated with different scenario of microenvironment reshaping.^37^

In the era of risk‐adapted treatment in HL, early identification of high‐risk patients is critical to address a personalized aggressive treatment.^38^ So far, PET‐2 is recognized as the most reliable tool to base a risk‐adapted strategy, to switch to intensified or de‐escalate treatment. However, PET‐2 result is a relatively late information, available at a time when mechanisms of chemotherapy resistance could be already initiated. In addition, its predictive value is suboptimal since a backbone of about 10% of PET‐2‐negative patients is still at risk of treatment failure.^9^, ^11^, ^12^, ^13^, ^14^, ^38^, ^39^

Several strategies have been attempted to single out at baseline high‐risk patients, despite a negative PET‐2. Since the number of tumor‐associated macrophages^23^ contribute to define histological subtypes in HL and parameters derived from lymphoma tissue analysis have prognostic meaning,^18^ several biomarkers and parameters, reflecting the activity of microenvironment, have been proposed in the last years, but they have been never included in a risk‐adapted strategy because of lack of reproducibility.^7^, ^20^ In this perspective, NLR and LMR, which represent a reproducible, cheap, and accessible biomarkers for all patients at diagnosis, could reflect the balance between the immunosuppressive capacity of myeloid cells and the number of lymphoid cells.

The clinical significance of NLR or LMR has been already described in the pre‐PET‐2 era (Table S1).^26^, ^29^ Koh et al found that NLR > 4.3 was associated with worse OS, but not EFS, in both early and advanced stage cHL.^29^ In a cohort of 312 early stage patients treated at MD Anderson Cancer Center high NLR was associated with worst FFP on univariate analysis, but only the platelet‐to‐lymphocyte count was an independent prognostic factor of relapse or refractory disease, without taking into account PET‐2 status.^30^ Similarly, Marcheselli et al found that NLR > 6 was associated with worse 5‐year PFS and OS in both early and advanced‐stage cHL.^26^

Other groups have investigated LMR as predictor of in HL (Table S1) in both single and multicenter large retrospective series, also in PET‐2 treatment‐adapted therapy settings, using different cutoffs: 1.1, 2, 2.1, 2.9. In the largest series of 1450 patients, LMR < 2.1 was associated with 5‐year PFS and OS of 74% and 88%, respectively.^32^ In another series of 121 patients, LMR < 2.1 was an independent predictor of PFS and OS,^36^ a controversial finding not confirmed in other series^28^ and in patients receiving a PET‐2‐adapted treatment.^6^, ^35^ LMR < 2.8 was predictor of lymphoma‐specific survival only in patients younger than 60 years in a large multicenter series including both early and advanced‐stage patients.^40^

Bari et al recently published that NLR, LMR, and IPS did not retain any predictive value in the HD0801 trial in which patients with a positive PET‐2 (defined as carrying Deauville score 3 or more) after two ABVD cycles underwent a more intense treatment with an early stem‐cell transplantation and four cycles of IGEV (ifosfamide, gemcitabine, vinorelbine, prednisone) regimen.^41^ Since IPS maintained its prognostic meaning in those trials in which the treatment of PET‐2 positive was switched from ABVD to escBEACOPP patients, the authors concluded that the intensification treatment with autologous transplantation in PET‐2‐positive patients could justify the disappearance of any relevant prognostic factor at baseline, including IPS and NLR. The discrepancy with our results could also be due to the different patient evaluation at PET‐2, since in the HD0801 patients with DS = 3 or more belonged to the PET‐2‐positive cohort, while in the HD0607 trial DS = 3 belonged to the PET‐2‐negative cohort, and the salvage regimen was more intensive in the study design of the HD0801 trial.

In the attempt to improve the prognostic meaning of IPS, Diefenbach et colleagues^42^ had shown that the lymphocyte component of the IPS7 had poor prognostic value, and proposed a score called IPS3 (age, hemoglobin, and stage) which was simpler and provided stronger prognostic information. In our series we could not validate IPS3 since the covariates, part of IPS, identified by our multivariable analysis of PFS or OS did not include age, hemoglobin, and stage.

We found that large nodal mass (>7 cm, in >=IIB stage patients) was associated with inferior outcome, but we did not perform a further subanalysis to investigate the contribution of the site. In contrast, Shunan et al have recently showed that mediastinal bulk (defined as large mediastinal mass of at least 5 cm) was associated with favorable course of the disease, in a retrospective cohort of 814 patients (stage III‐IV), treated in a pre‐PET‐2‐adapted treatment era.^43^

Different from prior analyses, our study was focused exclusively on advanced stage patients, included IPS and PET‐2 as major prognostic factors in multivariable analysis, and assessed the predictive role on treatment outcome of both NLR and LMR, disclosing that NLR, rather than LMR, can add at diagnosis prognostic information, especially if combined to IPS and the presence of large nodal mass. However, due to the low sensitivity and specificity, NLR alone cannot be considered for selecting treatment from the beginning since it is not such a robust biomarker that can identify with certainty patients with poor prognosis at diagnosis, without relying on PET‐2. NLR, with the stronger contribution of IPS ≥ 3, could help in identifying those few patients who relapse despite a PET‐2 negativity.

## AUTHOR CONTRIBUTIONS

AR, CP, FDR, AG, and AR conceptualized and designed the study. AR, CP, FDR, AG, CT, SV, and AR involved in data analysis and interpretation. All the authors made provision for study materials and patients, collected and assembled the data, involved in manuscript writing, gave the final approval of manuscript, and accountable for all the aspects of the work.

## Supporting information

Table S1‐5

## Data Availability

Raw data are available upon request to the corresponding author.
