# Supplementary material for: The neutrophil to lymphocyte ratio (NLR) and the presence of large nodal mass are independent predictors of early response: A subanalysis of the prospective phase II PET‐2‐adapted HD0607 trial
Source: Cancer Med. 2020 Nov 6;9(23):8735–46. doi: 10.1002/cam4.3396 (PMC7724487; doi:10.1002/cam4.3396)
Supplement: Supplementary file 1 — Table S1‐5 [file CAM4-9-8735-s001.pdf]

Supplementary Table 1

NLR and LMR in previously published series

# pts	Stage disease	Chemotherapy protocol(s)	NLR cut-off	PFS based on high NLR	OS based on high NLR	Study design	Ref.
180	Early+ advanced	ABVD ABVD-BEACOPP	6	5-yrs PFS 70.1%	Not reported	Retrospective Single-center	35
990	Early+ advanced	ABVD BEACOPP VBM MEC	6	5-yrs PFS 75%	5-yrs OS 88%	Retrospective multi-center	26
338	Early	Not specified	6.4	2-yrs FFP 82.2%	Not reported	Retrospective Single-center	30
312	Early+ advanced	ABVD MOPP ABVD/MOPP BEACOPP	4.3	NLR is not predictor of 5-yrs PFS	5-yrs OS 80.3%	Retrospective multi-center	29
**771**	**Advanced**	**ABVD ABVD-BEACOPP**	**6**	**3-yrs PFS 76%**	**NLR is not predictor of 3-yrs OS**	**Prospective multi-center PET-2 risk adapted**	**Present study**

# pts	Stage disease	Chemotherapy protocol(s)	LMR cut-off	PFS based on high LMR	OS based on high LMR	Study design	Ref.
180	Early+ advanced	ABVD ABVD-BEACOPP	2	5-yrs PFS 70.2%	Not reported	Retrospective Single-center	35
312	Early+ advanced	Not specified	2.9	5-yrs PFS 60.5%	5-yrs OS 77.5%	Retrospective multi-center	28
121	Early+ advanced	ABVD	2.1	5-yrs PFS 67.2%	5-yrs OS 74.3%	Retrospective multi-center	36
1450	Early+ advanced	ABVD BEACOPP VBM MEC	2.1	5-yrs PFS 74%	5-yrs OS 88%	Retrospective multi-center	32
351	Early+ advanced	ABVD	2.8	5-yrs LSS 84.3% (in pts<60 yrs)	5-yrs OS 80.3% (in pts<60 yrs)	Retrospective multi-center	43
537	Early+ advanced	ABVD	1.1	10-yrs TTP 48%	Not evaluated	Retrospective Single-center	34
101	Advanced	ABVD	2	5-yrs EFS 40%	5-yrs OS 54%	Retrospective Single-center	33
190	Early+ advanced	ABVD	1.1	5-yrs EFS 8%	5-yrs OS 27%	Retrospective Single-center	31
476	Early+ advanced	ABVD MOPP-ABV	1.1	5-yrs EFS 34%	5-yrs OS 52%	Retrospective Single-center	27
**771**	**Advanced**	**ABVD ABVD-BEACOPP**	**2**	**3-yrs PFS 79%**	**LMR is not predictor of 3-yrs OS**	**Prospective multi-center PET-2 risk adapted**	**Present study**

Supplementary Table 2 Median value and interquartile range of NLR and LMR at baseline in advanced-stage HL patients enrolled in the HD0607 trial, based on clinical characteristics and clinical outcome

Characteristics	NLR median (IQR)	p-value	LMR median (IQR)	p-value
**Age (years)**		*0.0004*		0.2667
<45	5.9 (4.0-8.7)		2.0 (1.4-2.8)	
≥45	4.8 (2.9-7.4)		2.1 (1.5-2.9)	
**Sex**		*0.0022*		*0.0142*
Female	6.0 (4.1-9.0)		2.1 (1.5-2.9)	
Male	5.4 (3.5-7.8)		1.9 (1.4-2.7)	
**Ann Arbor Stage**		0.4767		0.78
IIB	5.9 (4.0-8.4)		2.0 (1.4-2.8)	
III-IV	5.6 (3.7-8.3)		2.0 (1.4-2.8)	
**B Symptoms**		*<.0001*		*<.0001*
no	4.9 (3.3-6.5)		2.4 (1.7-3.3)	
yes	5.9 (4.0-9.0)		1.9 (1.4-2.7)	
**Bulky**		*<.0001*		*<.0001*
≤7 cm	5.1 (3.5-7.5)		2.1 (1.6-2.9)	
>7 cm	7.0 (4.5-10.1)		1.7 (1.3-2.5)	
**Bone marrow biopsy**		*0.0207*		*0.1629*
Negative	5.75 (3.89-8.4)		1.97 (1.42-2.79)	
Positive	4.33 (3.15-6.5)		2.53 (1.56-2.88)	
**IPS**		*<.0001*		*<.0001*
<3	5.2 (3.5-7.5)		2.2 (1.6-3.0)	
≥3	7.1 (4.8-11.0)		1.7 (1.2-2.4)	
**PET-2**		*<.0001*		0.12
Negative	5.5 (3.6-8.0)		2.0 (1.5-2.9)	
Positive	6.8 (4.7-10.7)		1.9 (1.3-2.5)	
**DS**		*0.0001*		*0.042*
0-3	5.5 (3.6-8.0)		2.0 (1.5-2.9)	
4	6.5 (4.3-10.5)		1.9 (1.2-2.5)	
5	7.3 (5.2-11.0)		1.9 (1.5-2.5)	

Abbreviations: NLR, neutrophil/lymphocyte ratio; LMR, lymphocyte to monocyte ratio; IQR, interquartile range; DS, Deauville score; IPS, International Prognostic Score.
Differences in continuous parameters were evaluated using Mann-Whitney U test. Significant values are in italic.

Supplementary Table 3 Univariate analysis of progression free survival in advanced-stage HL patients enrolled in the HD0607 trial, based on the main prognostic features, including PET2 status

Prognostic feature	N (%)	HR (95% CI)	p-value
**NLR**	771		
≤6	418 (54.2)	1	
>6	353 (45.8)	1.84 (1.31-2.58)	*0.0003*
**LMR**	771		
≤2	399 (51.8)	1	
>2	372 (48.2)	0.67 (0.48-0.95)	*0.0224*
**B Symptoms**	771		
Absent	147 (19.1)	1	
Present	624 (80.9)	1.42 (0.9-2.27)	0.1329
**Large nodal mass**	771		
≤7 cm	461 (59.8)	1	
>7 cm	310 (40.2)	1.36 (0.97-1.89)	0.0718
**IPS**	771		
<3	507 (65.8)	1	
≥3	264 (34.2)	2.5 (1.79-3.49)	*<.0001*
**PET-2**	769		
Negative	620 (80.6)	1	
Positive	149 (19.4)	3.89 (2.77-5.46)	*<.0001*

Abbreviations: 95% CI, 95% confidence interval; HR, Hazard ratio; NLR, neutrophil/lymphocyte ratio; LMR, lymphocyte to monocyte ratio; IPS, International Prognostic Score.
Significant P values are in italic.

Supplementary Table 4 Univariate and multivariable analysis of the main prognostic features at presentation

Predictive factors at baseline	N (%)	Univariate		Multivariable for NLR		Multivariable for LMR	
		HR (95% CI)	p-value	HR (95% CI)	p-value	HR (95% CI)	p-value
**NLR**	771						
≤6	418 (54.2)	1	1	1			
>6	353 (45.8)	1.95 (1.36-2.81)	*0.0003*	1.5 (1.01-2.21)	*0.0425*		
**LMR**	771						
≤2	399 (51.8)	1	1			1	
>2	372 (48.2)	0.69 (0.48-0.99)	*0.0476*			0.91 (0.62-1.34)	0.6221
**B Symptoms**	771						
Absent	147 (19.1)	1	1	1		1	
Present	624 (80.9)	0.97 (0.63-1.55)	0.9044	0.7 (0.43-1.14)	0.1403	0.73 (0.46-1.19)	0.1989
**Large nodal mass**	771						
≤7 cm	461 (59.8)	1	1	1		1	
>7 cm	310 (40.2)	2.25 (1.57-3.25)	*<.0001*	2.15 (1.48-3.16)	*0.0001*	2.31 (1.59-3.37)	*<.0001*
**IPS (N)**	771						
<3	507 (65.8)	1	1	1		1	
≥3	264 (34.2)	2.19 (1.52-3.15)	*<.0001*	2.18 (1.48-3.2)	*0.0001*	2.29 (1.55-3.37)	*<.0001*

Abbreviations: 95% CI, 95% confidence interval; HR, Hazard ratio; NLR, neutrophil/lymphocyte ratio; LMR, lymphocyte to monocyte ratio; IPS, International Prognostic Score.
Significant P values are in italic.

Supplementary Table 5 Univariate and multivariable analysis of progression free survival in 620 PET-2 negative HL patients enrolled in the HD0607 trial, based on the main prognostic features at presentation

Predictive factors at baseline	N (%)	Univariate		Multivariable for NLR		Multivariable for LMR	
		HR (95% CI)	P	HR (95% CI)	P	HR (95% CI)	P
**NLR**	620						
≤6	356 (57.4)	1		1			
>6	264 (42.6)	1.57 (1.01-2.44)	*0.0432*	1.34 (0.84-2.14)	0.2152		
**LMR**	620						
≤2	310 (50)	1				1	
>2	310 (50)	0.72 (0.46-1.12)	0.1476			0.86 (0.54-1.36)	0.5157
**B Symptoms**	620						
Absent	118 (19)	1		1		1	
Present	502 (81)	1.4 (0.76-2.58)	0.2857	1.15 (0.62-2.16)	0.6552	1.19 (0.63-2.22)	0.5918
**Large nodal mass**	620						
≤7 cm	394 (63.5)	1		1		1	
>7 cm	226 (36.5)	1.21 (0.77-1.89)	0.4126	1.14 (0.72-1.81)	0.5816	1.19 (0.75-1.88)	0.4573
**IPS**	620						
<3	431 (69.5)	1		1		1	
≥3	189 (30.5)	1.98 (1.27-3.08)	*0.0024*	1.85 (1.18-2.92)	*0.0078*	1.89 (1.2-2.99)	*0.0061*

Abbreviations: 95% CI, 95% confidence interval; HR, Hazard ratio; NLR, neutrophil/lymphocyte ratio; LMR, lymphocyte to monocyte ratio; IPS, International Prognostic Score.
Significant P values are in italic.
